# Supplementary material for: The interactome of intact mitochondria by cross-linking mass spectrometry provides evidence for coexisting respiratory supercomplexes
Source: Mol Cell Proteomics. 2017 Dec 8;17(2):216–32. doi: 10.1074/mcp.RA117.000470 (PMC5795388; doi:10.1074/mcp.RA117.000470)
Supplement: Supplemental Data [file supp_RA117.000470_133922_0_supp_23978_fzffwf.zip › spectra_annotation/mito_DR_spectra_annotation/77-1-3-1-7-1.pdf]

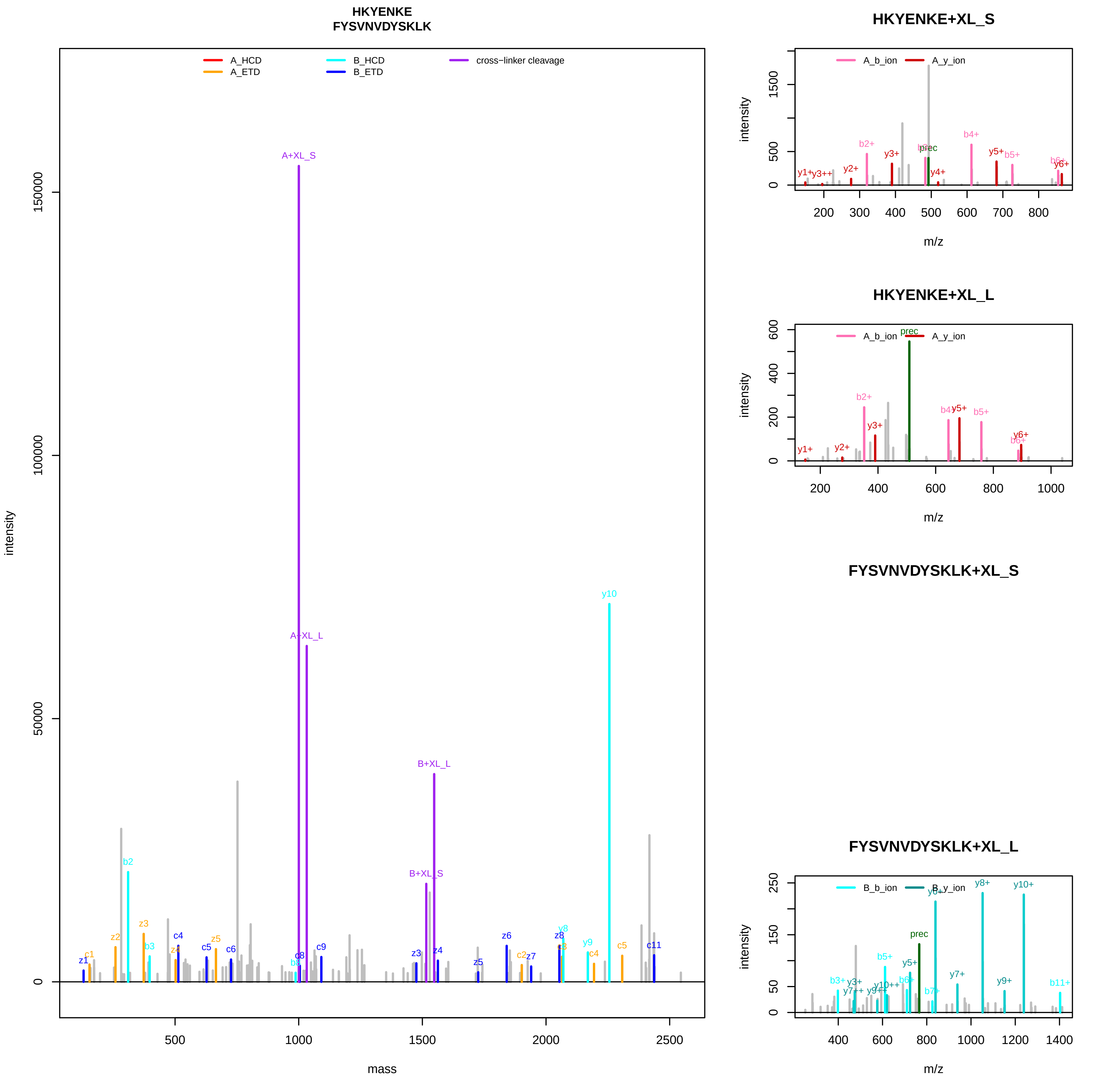
HKYENKE
FYSVNVDYSKLK
A_HCD
A_ETD
B_HCD
B_ETD
cross−linker cleavage
A+XL_S
A+XL_L
B+XL_L
B+XL_S
y10
b2
z1
c1
z2
z3
b3
z4
c4
c5
z5
c6
c8
b8
c9
z3
z4
z5
z6
c2
z7
z8
y8
c3
y9
c4
c5
c11
intensity
mass
0
50000
100000
150000
500
1000
1500
2000
2500
HKYENKE+XL_S
A_b_ion
A_y_ion
b2+
y3+
prec
b4+
y5+
b5+
y1+
y3++
y2+
y4+
b6+
y6+
intensity
m/z
0
500
1500
200
300
400
500
600
700
800
HKYENKE+XL_L
A_b_ion
prec
A_y_ion
b2+
y3+
b4+
y5+
b5+
y6+
b6+
y1+
y2+
intensity
m/z
0
200
400
600
200
400
600
800
1000
FYSVNVDYSKLK+XL_S
FYSVNVDYSKLK+XL_L
B_b_ion
B_y_ion
y8+
y10+
prec
b5+
y5+
b3+
y3+
y7++
y9++
y10++
b6+
y7+
y9+
b7+
b11+
intensity
m/z
0
50
150
250
400
600
800
1000
1200
1400
